# A study on pregenomic RNA and factors in the pregnant and postpartum women with chronic HBV infection based on real world

**DOI:** 10.3389/fcimb.2025.1539356

**Published:** 2025-04-04

**Authors:** Juan Tang, Qiuchen Wu, Ziyue Zhang, Guanlun Zhou, Ying Ji, Yandan Wu, Genju Wang

**Affiliations:** ^1^ Department of Obstetrics and Gynecology, The Second Hospital of Nanjing, Affiliated to Nanjing University of Chinese Medicine, Nanjing, Jiangsu, China; ^2^ Department of Microbiology and Immunology, Medical School of Southeast University, Nanjing, Jiangsu, China

**Keywords:** chronic hepatitis b, pregnancy, postpartum, pgRNA, infection

## Abstract

**Introduction:**

Most studies have focused on mother-to-child transmission and postpartum hepatitis flares. We aimed to evaluate the expression profile of pregenomic RNA (pgRNA) and its associated factors in patients with chronic HBV infection (CHB) during pregnancy and postpartum.

**Methods:**

A total of 134 pregnant and 100 postpartum CHB patients were enrolled, and serum pgRNA levels were quantified.

**Results:**

Significant differences in clinical characteristics were noted between pregnant and postpartum CHB patients, but there was no significant difference in pgRNA levels between the two groups. When HBV DNA and HBV antigen levels were low, the corresponding pgRNA detection rate decreased. pgRNA was positively correlated with DNA and HBV antigens in the pregnant and postpartum CHB patients. In the pregnant group, antiviral treatment was an independent risk factor for pgRNA levels. In the postpartum period, HBeAg levels and antiviral treatment were independent risk factors for pgRNA levels. Considering that patients receiving antiviral treatment often have a high DNA load or HBeAg positivity, the DNA level and HBeAg essentially affect pgRNA levels.

**Conclusion:**

pgRNA levels differ between pregnant and postpartum patients, and this difference is of great significance for the diagnosis and management of these particular groups.

## Introduction

Despite the availability of vaccines, hepatitis B virus (HBV) infection still rages globally, affecting more than 250 million people. In China, mother-to-child transmission is the main route of HBV transmission, specifically intrauterine infection, infection during the delivery process, and postpartum infection (Jing et al., 2020). Pregnant women with chronic hepatitis B do not need to be vaccinated against hepatitis B. Instead, their viral load needs to be evaluated to determine whether antiviral treatment is needed. For pregnant women who are HBeAg-positive or have a high viral load, preventive antiviral interventions are required according to current guideline recommendations (European Association for the Study of the Liver, 2017; Terrault et al., 2018; You et al., 2023). Moreover, newborns should receive hepatitis B vaccine and hepatitis B immunoglobulin in a timely manner after birth to minimize the risk of mother-to-child transmission. For pregnant women who are positive for HBeAg or have a high viral load, preventive antiviral interventions are needed according to current guideline recommendations (European Association for the Study of the Liver, 2017; Terrault et al., 2018; You et al., 2023). Of note, chronic hepatitis B virus (HBV) infection is a dynamically developing disease, and its remarkable feature involves the intricate interactions among HBV, hepatocytes, and the host immune system. During pregnancy, in order to establish immune tolerance in the fetus, a series of changes in the maternal immune system occur, providing overall enhanced immune tolerance. Maternal cell-mediated immunity is suppressed during pregnancy to maintain an immune-tolerant state and prevent the fetus from being rejected by the maternal immune system. The activity of natural killer cells is reduced, T-lymphocyte function is restricted, and Th1-type cytokine secretion decreases, resulting in a decline in antiviral immune capacity (Lu et al., 2023). This immunosuppressive state will reverse and the immune system rapidly rebounds during the postpartum. This change is likely to exacerbate the liver inflammatory response, causing fluctuations in of chronic HBV infection and thus creating complex challenges for the prevention and treatment of HBV infection (Zhang et al., 2022).

During the life cycle of the hepatitis B virus, HBV cccDNA (covalently closed circular DNA) in hepatocytes serves as the most important template for the coding of HBV RNA. HBV cccDNA is not only involved in the initiation of viral replication but also a key factor in persistent infection and relapse (Zhang et al., 2019). Current therapies have been shown to have minimal effect on cccDNA, the long-term presence of which is an important cause of chronic HBV (Vachon and Osiowy, 2021; Yan et al., 2024). Theoretically, cccDNA levels should be the most direct indicator of prognosis (Xia and Guo, 2020). However, the application of cccDNA is limited because of its uneven distribution and the damage caused by invasive tests (Dong et al., 2018).In contrast, HBV pgRNA (pregenomic RNA) is a simple and noninvasive test, and is therefore more acceptable to patients (Yan et al., 2023; Yu et al., 2022). pgRNA is a direct transcript of cccDNA. Studies have shown that pgRNA is positively correlated with cccDNA, providing important clues regarding the status of cccDNA activity (Shen et al., 2020). HBV pgRNA is detected at significantly lower levels than HBV DNA in the serum of patients with chronic hepatitis B. Serum HBV pgRNA is an important biomarker that reflects the persistence of hepatitis B virus infection, the replication status of hepatitis B virus, and the transcriptional activity of intrahepatic covalent closed-loop DNA in the liver tissues of chronic hepatitis B patients. In addition, serum HBV has been used as an early predictor of antiviral therapy, a marker of the emergence of hepatitis B virus resistance, and as an indicator of safe discontinuation of nucleoside analogue therapy (Carey et al., 2020; Fan et al., 2020a, 2020; Luo et al., 2022). However, most of the current clinical studies on pgRNA have focused on nonpregnant patients with chronic hepatitis B. Only four reports have described the clinical utility of pgRNA in pregnant women with chronic HBV (Patel et al., 2019; Wang C. et al., 2022; Wang C. R. et al., 2022; Wang et al., 2024). Wang et al. reported that among pregnant women with HBeAg-positive chronic hepatitis B receiving antiviral therapy, those who achieved serologic conversion of HBeAg and a decrease in HBsAg exhibited faster decreases in pgRNA and HBcrAg levels. In addition, a postpartum pgRNA decline and an ALT peak predict HBsAg decline after postpartum drug withdrawal in pregnant women with chronic hepatitis B (Wang C. R. et al., 2022). However, these study cohorts were relatively small and lacked a comprehensive assessment of pgRNA in these specific groups, namely, the pregnant and postpartum groups. This study aimed to compare the expression characteristics of pgRNA and its influencing factors in CHB patients during pregnancy and postpartum.

## Methods

### Patients

A total of 134 women with chronic HBV infection during pregnancy and 100 women with chronic HBV infection in the postpartum period who attended the Second Hospital of Nanjing from September 2022 to June 2023 were included. Pregnant and postpartum women with chronic HBV were included, regardless of whether they received treatment during pregnancy or the postpartum period. The clinical data of the patients are presented in **Table 1**. The inclusion criteria were as follows: 1) pregnant and postpartum female patients aged 20-49 years; 2) patients who were HBsAg positive for more than 6 months; and 3) patients with clinical, biochemical, and viral symptoms of chronic HBV infection. Among them, 20 patients were in the first trimester (less than 14 weeks of gestation), 63 patients were in the second trimester (14-28 weeks of gestation), and 51 patients were in the third trimester (28-40 weeks of gestation). In the postpartum period, there were 20 patients within 0-3 months of giving birth, 22 patients within 3-12 months, 58 patients within 12-18 months. The exclusion criteria were as follows: 1) hepatitis A, hepatitis C, human immunodeficiency virus, or other viral infections; 2) other chronic liver diseases (autoimmune liver disease, alcoholic liver disease, fatty liver disease, etc.); and 3) cirrhosis, hepatocellular carcinoma, or obstetric disorders (e.g., intrahepatic cholestasis during pregnancy). This retrospective analysis of data collected from patients’ daily testing programs and clinical information, was approved by the Clinical Ethics Committee of the Second Hospital of Nanjing (2024-LS-ky099), and all enrolled patients were exempted from signing an informed consent form.

**Table 1:** Clinical virological characteristics of CHB patients included in the study.

Characteristics		Pregnant (n = 134) Median (min-max)	Postpartum (n = 100) Median (min-max)	M-W/P
**Age (years)**		31 (19-39)	34 (20-45)	0.890
**ALT (IU/L)**		15.6 (6.8-89.72)	18.45 (7.3-166.3)	0.006
**AST (IU/L)**		16.45 (1.09-97.2)	20 (11.3-109.6)	**<0.0001**
**AFP (ng/mL)**		13.6 (0-147.7)	2.2(0.85-7.44)	**<0.0001**
**pgRNA (log copies/mL)**		2.96(<2-8.36)	2.99(<2-8)	0.450
**HBV DNA (log IU/mL)**		3.16(<1.30-9)	1.30 (<1.30-8.23)	**<0.0001**
**HBsAg (IU/mL)**		4390.74 (0.19->52000)	2526.92 (0.01->52000)	0.670
**HBeAg (COI)**		9.867 (0.01-1971)	1.37 (0.01-1814.34)	0.840
**HA (ng/mL)**		44.6 (30-624)	53.1 (30-289.5)	**0.043**
**PIIINP (ng/mL)**		9.80 (2.17-30.36)	8.08 (3.38-29.16)	0.0012
**LN (ng/mL)**		75.7 (8.2-160.7)	80.4 (10.2-166.2)	0.110
**CIV (ng/mL)**		64.9 (6.69-123.9)	49.9 (1-98.4)	<0.0001
**Treatment (patients)**	Treated-NUCs	34	43	
	Treated-NUCs + IFN	0	29	
	Untreated	100	28	
**Weeks of pregnancy**		20(8-40)		
**Months after postpartum**			12(0.5-18)	

M-W, Mann-Whitney test.Bold values indicate p < 0.05.

### pgRNA detection

pgRNA was detected using the RNA simultaneous amplification testing method (HBV-SAT) via real-time fluorescence detection of isothermal RNA amplification with an HBV-SAT kit (Shanghai Rendu Biotechnology Co., Ltd. China) according to the manufacturer’s instructions. Briefly, the RNA of the samples was extracted using magnetic microparticles with HBV specific RNA oligonucleotides. The target RNA was reverse transcribed using the MMLV enzyme, transcribed with T7 RNA polymerase and detected using an RNA molecular beacon probe labeled with fluorescence and a quencher. RNA extraction, amplification, and detection were performed using an automated AutoSAT system (Shanghai Rendu Biotechnology Co., Ltd. China). An internal calibrator/internal control (IC) was added to every single reaction. The concentration of a sample was determined using the HBV and IC signals for each reaction and compared with the calibration information. The linear range was established by testing panels of HBV RNA diluted in HBV-negative human serum. The concentration ranged in concentration from 2 log copies/mL to 8 log copies/mL. The R^2^ value of the linear equation is greater than 0.98. The limit of detection (LOD) is 50 copies/mL. The limit of quantitation (LOQ) is 100 copies/mL.

### Clinical indicator detection

The levels of alanine aminotransferase (ALT), aspartate aminotransferase (AST), HBsAg, and HBeAg were measured with a Roche Cobas 8000 automated biochemical immunoassay analyzer. The blood samples were processed by the instrument according to the manufacturer’s procedures. Briefly, for ALT and AST, specific reagents were used to react with the respective substances in the sample. Changes in absorbance were measured to calculate ALT and AST concentrations. ALT and AST levels less than 40 IU/L were considered negative. For HBsAg and, HBeAg, specific antibodies and antigens were used to bind to the target substances in the sample. The results were obtained through a series of immunoassay reactions. Here, HBsAg levels < 0.05 IU/mL were considered negative and HBeAg < 1 COI was considered negative. For quantitative detection of the HBV DNA load, a fluorescent quantitative polymerase chain reaction (PCR) method and the Roche Cobas 6800/8800 detection system were used. Briefly, DNA was extracted from a sample using a specific method, and the target DNA was amplified in a PCR reaction using specific primers and probes. The fluorescent signal generated during the amplification process was detected and analyzed. HBV DNA concentration was determined by comparing the fluorescence signal to calibration information. HBV DNA <20 IU/mL was considered negative. Alpha-fetoprotein (AFP) was detected by the Roche Cobas e601 automatic immunoanalyzer. AFP levels less than 10 ng/mL were considered negative. A liver fibrosis marker assay kit (Tellgen) was used to quantify the concentrations of laminin(LN), procollagen type III N - terminal peptide(PIIINP), type IV collagen(CIV), and hyaluronic acid(HA) in the serum using a TESMI F4000 fully automatic flow cytometry fluorescence luminescence immunoassay analyzer. LN concentrations less than 130 ng/mL, PIIINP concentrations less than 15 ng/mL, C IV concentrations less than 95 ng/mL, and HA concentrations less than 120 ng/mL were considered negative.

### Statistical methods

Statistical analysis was performed using GraphPad Prism 9. Count data are expressed as case numbers and rates (%). The chi-square test was used for comparisons between groups. Data are expressed as medians (interquartile ranges). The median pgRNA, HBV DNA, HBsAg, HBeAg, ALT, AST, AFP, LN, PIIINP, CIV, and HA levels between the two groups were analyzed using the Mann-Whitney (nonparametric) test. The Kruskal-Wallis test (nonparametric) was performed when more than two groups were assessed. Spearman correlation tests were performed to analyze correlations between continuous data. Logistic regression analysis was used to explore the factors influencing pgRNA levels. P < 0.05 was considered statistically significant.

## Results

### Differences in the clinical characteristics of pregnant and postpartum in patients with chronic hepatitis B

**Figure 1:**
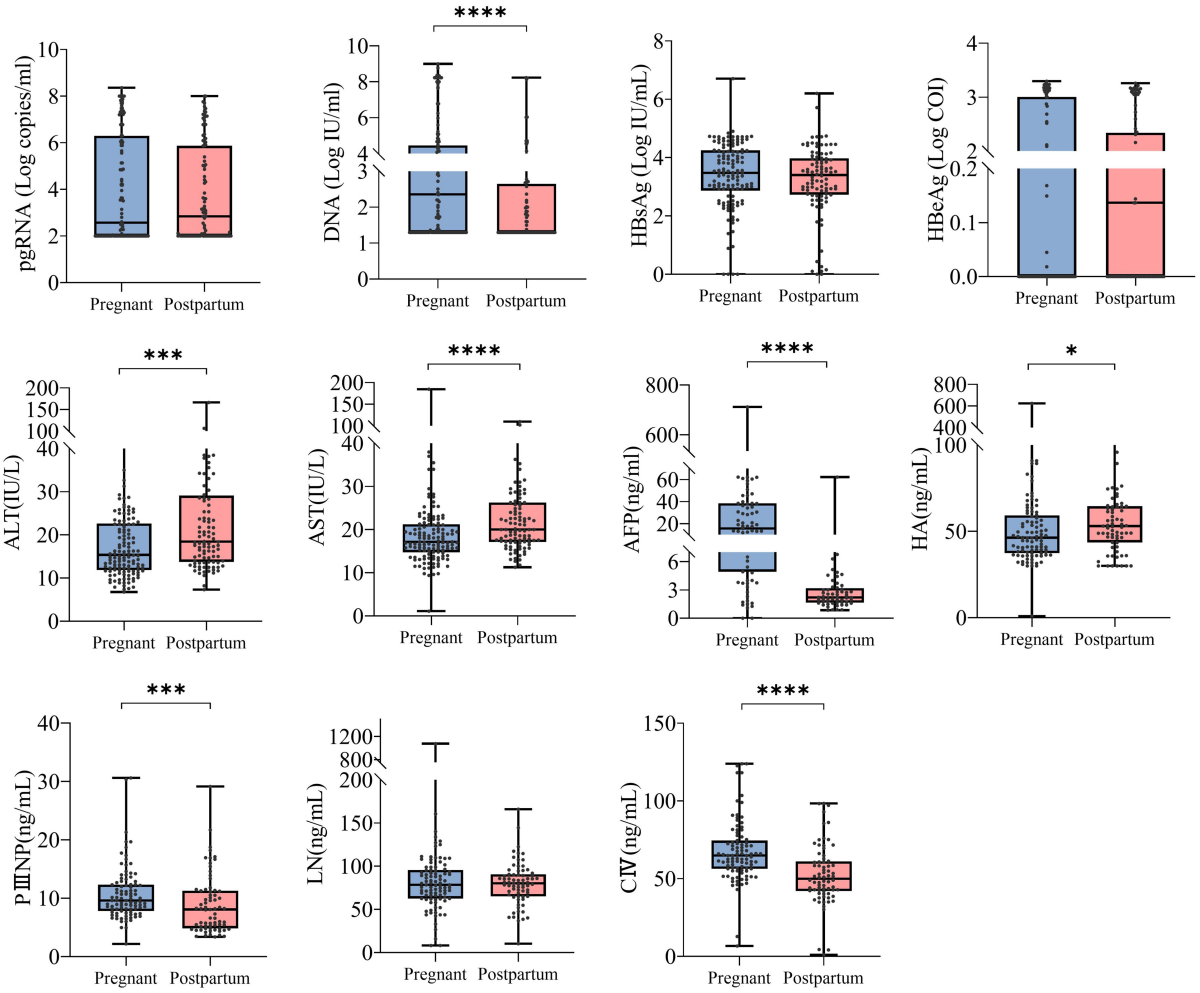
Differences in clinical characteristics between pregnancy and postpartum in patients with chronic hepatitis B. 234 CHB patients were grouped into pregnant (n =134) and postpartum groups (n =100). Then, HBV pgRNA, DNA, HBsAg, HBeAg, ALT, AST, AFP, LN, PIIINP, CIV, and HA were compared across the groups. The medians (interquartile range) are presented and statistical analyses were performed using Mann-Whitney test (M-W) between two groups. Asterisks indicate significance, *p < 0.05; ***p < 0.001; ****p < 0.0001.

This study was conducted to analyze 234 female patients with chronic hepatitis B, including 134 pregnant and 100 postpartum patients. The results revealed significant differences in several clinical characteristics between pregnant and postpartum patients. Specifically, HBV DNA and AFP levels were significantly higher in pregnant patients than in postpartum patients, whereas alanine aminotransferase (ALT) and aspartate aminotransferase (AST) levels were significantly higher in postpartum patients than in the pregnant patients. In addition, in terms of the four indicators of liver fibrosis (LN, PIIINP, CIV and HA), pregnant patients showed the differences with postpartum patients. However, when the treatment regimen, immune status of the host, and other factors were not taken into consideration, no significant difference was observed in the pgRNA levels between these two groups (**Figure 1**). In conclusion, patients with chronic hepatitis B exhibit distinct clinical characteristics between the two stages of pregnancy and the postpartum period.

### pgRNA detection rates in pregnant and postpartum patients with chronic hepatitis B

Consequently, we compared pgRNA detection rates in the pregnant (n = 134) and postpartum (n = 100) groups, which were divided according to clinical indicators (**Table 2**). In the pregnant group, significant differences in pgRNA detection rates were noted according to the treatment regimen, and the HBeAg, DNA, HBsAg, and AFP subgroups. Among them, the detection rate of pgRNA in the group receiving nucleoside analogue (NUCs) treatment (90.91%) was significantly greater than that in the untreated group (29.70%). This may be because patients receiving NUCs treatment have relatively high levels of HBeAg and DNA. On the other hand, the NUCs and HBV RNA detection rates were not correlated in this study. The detection rate was greater in the HBeAg-positive group (83.05%) than in the negative group (40.30%); and the detection rate in the group with high DNA levels (log IU/mL >5.0) (96.30%) was higher than that of the intermediate DNA level group (81.25%) (3.0 < log IU/mL <5.0) and low DNA level group (37.84%) (log IU/mL <3.0).The detection rate in the high HBsAg level (>10000 IU/mL) group (84.44%) was greater than that of the intermediate level (1000-10000 IU/mL) group (48.89%) and low level (<1000 IU/mL)group (40.00%).The detection rate of normal AFP group (75.00%) was greater than that of abnormal AFP group (46.81%). In the postpartum group, the pgRNA detection rate varied according to the treatment regimen, namely, HBeAg and HBsAg groups. The pgRNA detection rate was 34.48% in the group treated with pegIFN-α and NUCs, 79.07% in the group treated with NUCs, and 60.71% in the untreated group. pgRNA levels in patients in the combined treatment group were significantly lower than that in the other two groups, suggesting that pegIFN-α has the potential to reduce pgRNA levels. The detection rate in the HBeAg-positive group (97.87%) was greater than that in the negative group (26.67%); and the detection rate in the high HBsAg level group (>10000 IU/mL) was 100.00%, which was greater than that in the intermediate HBsAg level group (66.67%) (1000-10000 IU/mL) and low HBsAg level group (33.33%) (<1000 IU/mL). Compared with those in the pregnant group, the positive rates of pgRNA in the untreated postpartum group, the postpartum group with a low DNA load (3.0 < Log IU/mL <5.0), and the positive HBeAg group were significantly lower. These results suggested that different clinical characteristics may influence pgRNA detection. In addition, we focused on 106 patients with chronic hepatitis B (49 pregnant and 57 postpartum patients) whose HBV DNA levels were less than 20 IU/ml, and HBV pgRNA was detected in 41.5% (18 pregnant and 26 postpartum patients) of the patients. These findings suggest that when the HBV DNA level is below the limit of detection, the serum HBV pgRNA level can serve as an alternative marker for clinical monitoring, and provide a reference for selecting the appropriate time to stop antiviral therapy and adjusting the treatment regimen.

**Table 2:** Analysis of pgRNA Positive Rates in Pregnant and Postpartum Patients with Chronic Hepatitis B.

Pregnant (n = 134)					Postpartum (n = 100)				Pregnant vs Postpartum p-value
		positive cases/total number	Positive rate	p-value		positive cases/total number	Positive rate	p-value	
**Treatment**	treated-NUCs	30/33	90.91%	**<0.0001**	treated-NUCs	34/43	79.07%	**<0.001**	0.210
	untreated	30/101	29.70%		untreated	17/28	60.71%		**0.004**
					treated-NUCs + IFN	10/29	34.48%		
**DNA** (Log IU/ml)	low	28/74	37.84%	**<0.0001**	low	56/94	59.57%	0.408	**0.008**
	middle	26/32	81.25%		middle	10/15	66.67%		0.290
	high	26/27	96.30%		high	15/20	75.00%		0.070
**HBeAg** (COI)	positive	49/59	83.05%	**<0.0001**	positive	46/47	97.87%	**<0.0001**	**0.020**
	negative	27/67	40.30%		Negative	12/45	26.67%		0.220
**HBsAg** (IU/mL)	low	16/40	40.00%	**<0.0001**	low	12/36	33.33%	**<0.0001**	0.630
	middle	22/45	48.89%		middle	24/36	66.67%		0.120
	high	38/45	84.44%		high	23/23	100.00%		0.080
**ALT**(IU/L)	<40	73/125	58.40%	0.420	<40	52/87	59.77%	0.483	0.880
	>40	7/9	77.78%		>40	9/12	75.00%		1
**AST**(IU/L)	<40	79/131	60.31%	0.720	<40	56/92	60.87%	0.880	1
	>40	1/3	33.33%		>40	5/7	71.43%		1
**AFP** (ng/mL)	<10	24/32	75.00%	**0.023**	<10	38/51	74.51%	1	1
	>10	22/47	46.81%		>10	1/1	100.00%		1
**Four tests for liver fibrosis**	Abnormal	12/19	63.16%	0.863	Abnormal	12/15	80.00%	0.193	0.450
	Normal	62/117	52.99%		Normal	49/84	58.33%		0.470

Bold values indicate p < 0.05.

### Clinical characterization of pregnant and postpartum CHB patients with different pgRNA levels

**Figure 2:**
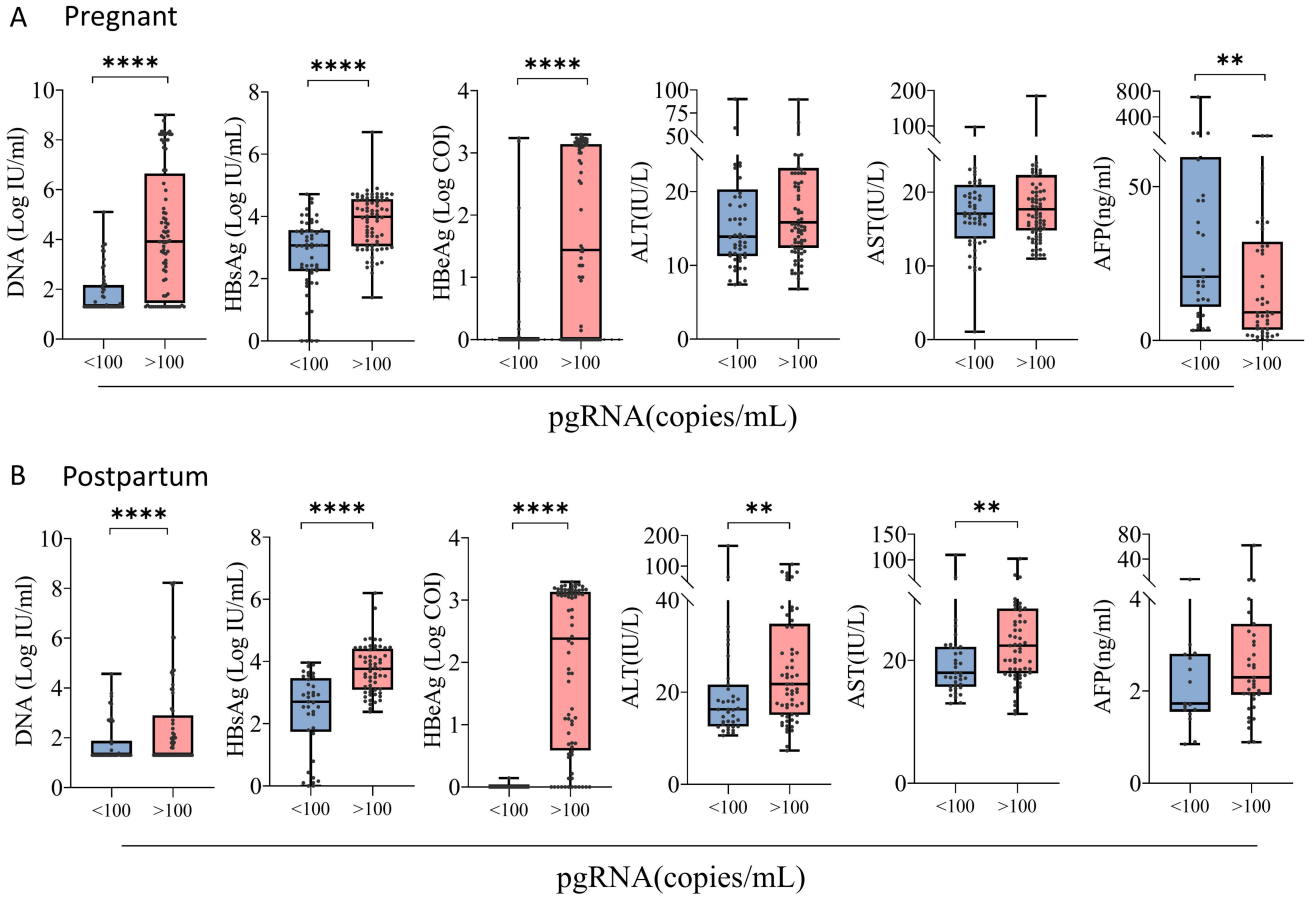
Clinical characterization of pregnant and postpartum CHB patients with different pgRNA levels. **(A)** A cohort of pregnant patients with hepatitis B virus were grouped by pgRNA levels. Then, the HBV DNA (<100, n = 53; >100, n = 80), HBsAg level (<100, n = 54; >100, n = 76), HBeAg level (<100, n = 50; >100, n = 76) and AFP level (<100, n = 33; >100, n = 46) were compared across the groups. **(B)** A cohort of postpartum patients with hepatitis B virus were grouped by pgRNA levels. Then, the HBV DNA (<100, n = 39; >100, n = 61), HBsAg level (<100, n = 37; >100, n = 59), HBeAg level (<100, n = 34; >100, n = 58) and AFP level (<100, n = 17; >100, n = 39) were compared across the groups. The medians (interquartile range) are presented and statistical analyses were performed using Mann-Whitney test (M-W) between two groups. Asterisks indicate significance, **p < 0.01; ****p < 0.0001.

Patients were categorized into positive (>100 copies/mL) and negative (<100 copies/mL) subgroups on the basis of pgRNA levels, and comparisons of laboratory indices were conducted. In the pregnancy group, DNA, HBsAg, HBeAg, and AFP levels differed according to in different pgRNA status. Specifically, pgRNA-positive patients tended to have higher levels of DNA, HBeAg, and HBsAg, whereas AFP was more highly expressed in pgRNA-negative patients (**Figure 2A**). In the postpartum group, differences in DNA, HBsAg, HBeAg, ALT, and AST were detected (**Figure 2B**). In terms of DNA, HBsAg and HBeAg, postpartum patients presented the same trends as pregnant patients did, with high levels of virologic indicators observed in pgRNA-positive patients. ALT and AST levels were significantly greater in pgRNA-positive postpartum patients than in negative patients, whereas no significant difference was detected in the pregnant group (**Figure 2**).

### pgRNA in pregnant and postpartum CHB patients with different seroviral profiles

**Figure 3:**
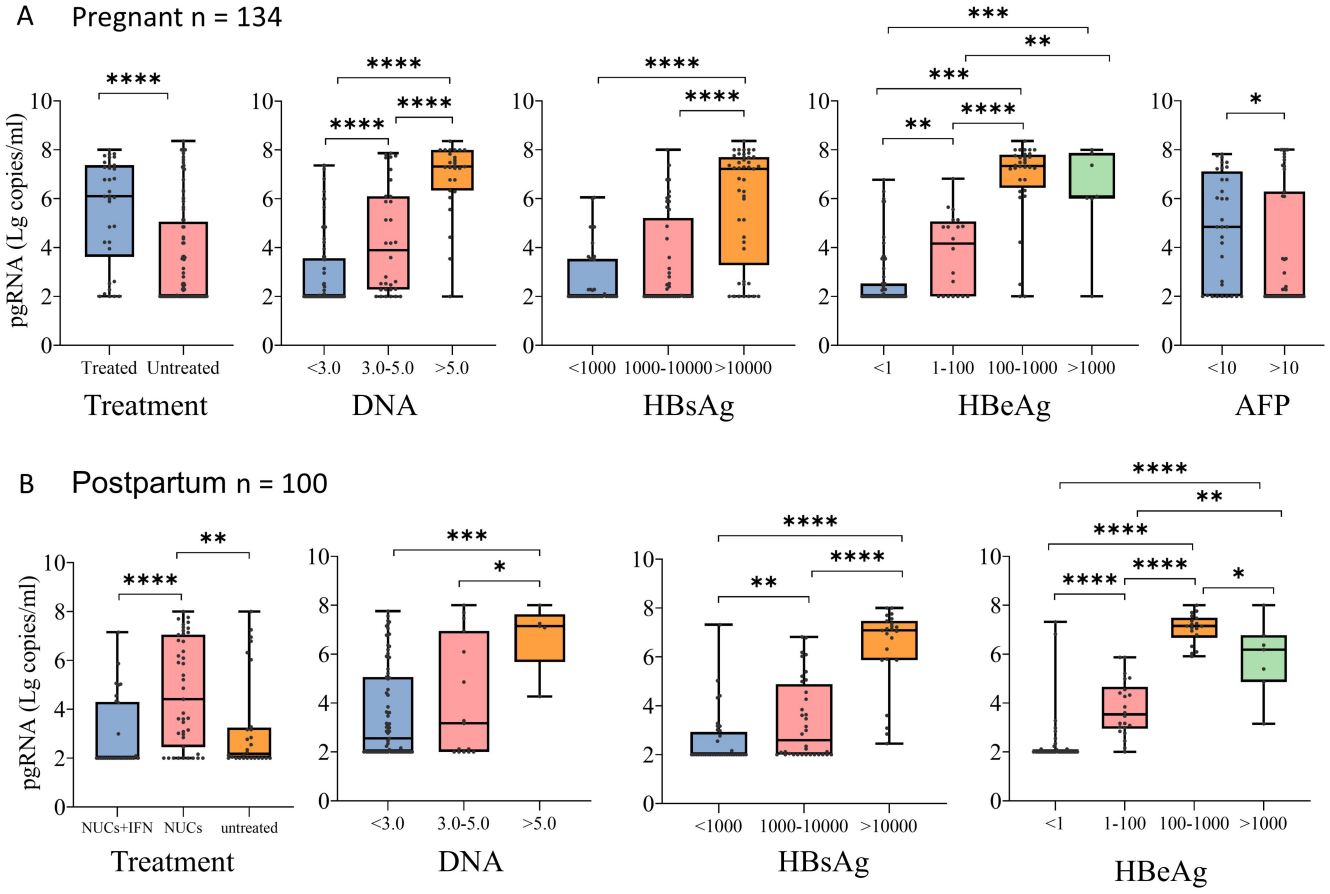
pgRNA in pregnant and postpartum CHB patients with different seroviral profiles. **(A)** A cohort of pregnant patients with hepatitis B virus were grouped by treatment (Treated, n = 34,Untreated = 100), HBV viral load (<3.0, n = 74; 3.0-5.0, n = 32; >5.0, n = 27), HBsAg level (<1000, n = 40; 1000-10000, n = 45; >10000, n = 45), HBeAg level (<1, n = 67; 1-100, n = 20; 100-1000, n = 33; >1000, n = 7) and AFP level (<10, n = 32; >100, n = 47). **(B)** A cohort of postpartum patients with hepatitis B virus were grouped by treatment (treated-IFN+NUCs, n = 29,treated-NUCs = 43, untreated = 28), HBV viral load (<3.0, n = 79; 3.0-5.0, n = 15; >5.0, n = 5), HBsAg level (<1000, n = 36; 1000-10000, n = 36; >10000, n = 23), and HBeAg level (<1, n = 45; 1-100, n = 22; 100-1000, n = 18; >1000, n = 7). Then pgRNA levels were compared across the groups. The medians (interquartile range) are presented and statistical analyses were performed using Mann-Whitney test (M-W) between two group. Asterisks indicate significance, *p < 0.05; **p < 0.01; ***p < 0.001; ****p < 0.0001.

Stratified analysis revealed that among 134 patients with chronic HBV infection during pregnancy, 34 patients were treated with NUCs (32 patients were treated with tenofovir disoproxil fumarate (TDF), and 2 patients were treated with tenofovir alafenamide (TAF)). The pgRNA levels were significantly higher in pregnant patients treated with NUCs than in the untreated patients, and the pgRNA level increased as the levels of DNA, HBsAg, and HBeAg increased (**Figure 3A**). No significant difference in pgRNA was detected between the normal and abnormal ALT and AST groups (**Supplementary Figure S1A**). The pgRNA levels were highest in 43 of the 100 postpartum patients treated with NUCs (10 treated with TAF and 33 treated with TDF), whereas the pgRNA levels were significantly lower in patients treated with a combination of pegIFN-α and NUCs (29 patients) than in patients treated with NUCs alone. This finding indicated that pegIFN-α reduces pgRNA levels in patients to some extent (**Figure 3B**). Similarly, pgRNA levels showed specific trends among different DNA, HBsAg and HBeAg subgroups (**Figure 3B**), whereas no significant differences in pgRNA levels were detected between the normal and abnormal groups for AFP, ALT, and AST (**Supplementary Figure S1B**). Notably, in pregnant patients, pgRNA was significantly lower in patients with low levels of DNA (Ig IU/mL < 3.0) than in patients with intermediate levels of DNA (3.0 < Ig IU/mL < 5.0). However, this difference was not detected in the postpartum group. In the postpartum group, pgRNA levels were significantly lower in patients with low levels of HBsAg (<1000 IU/mL) than in those with intermediate levels of HBsAg (1000-10000 IU/mL); this difference was not detected in the pregnant group.

### Correlation of pgRNA with clinical indicators in pregnant and postpartum patients with chronic hepatitis B

**Figure 4:**
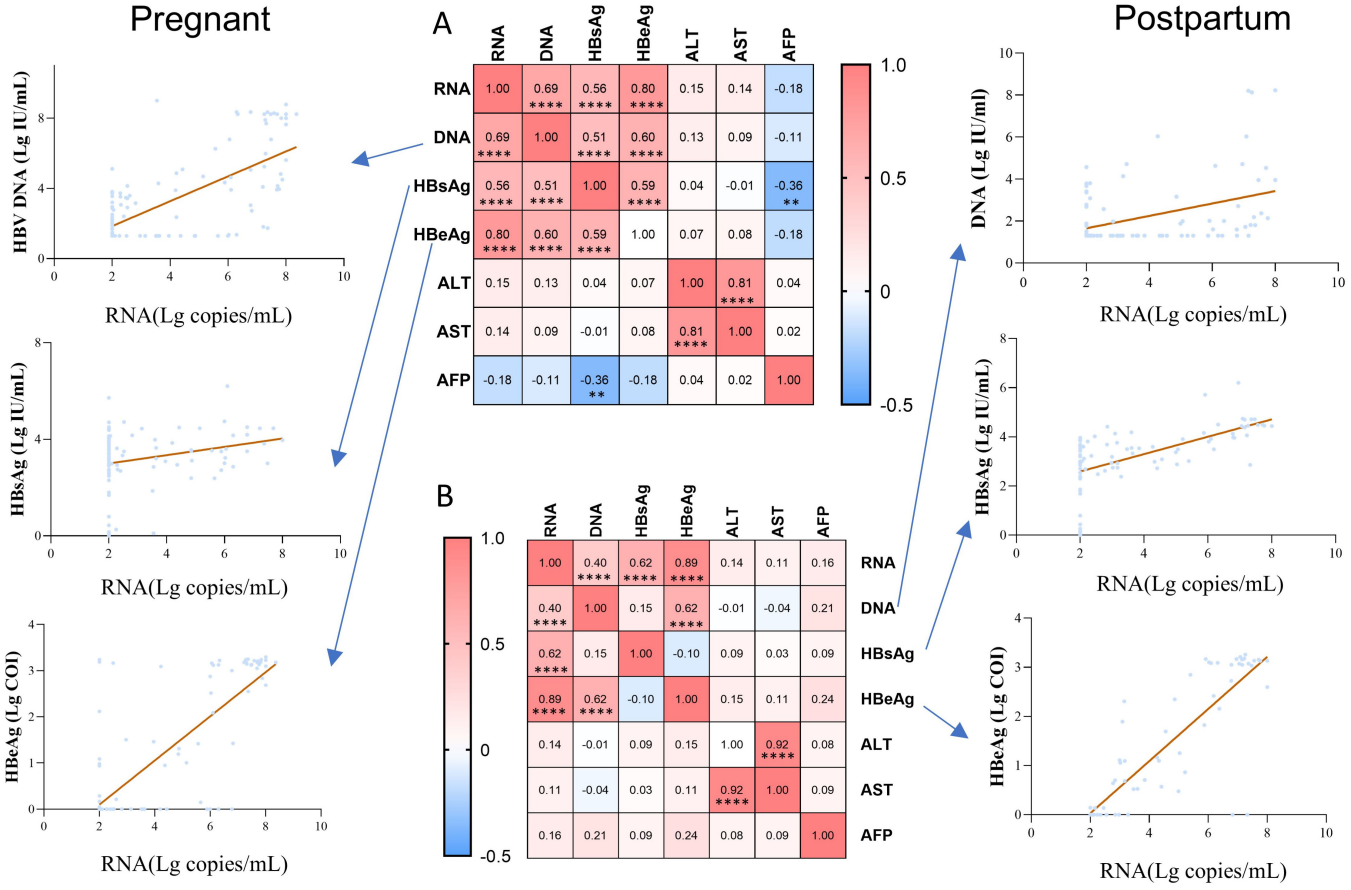
Correlation of pgRNA with clinical indicators in pregnant and postpartum patients with chronic hepatitis B. **(A)** Association between pgRNA and other markers in the pregnant patients with hepatitis B virus. **(B)** Association between pgRNA and other markers in the postpartum patients with hepatitis B virus. The correlation coefficients between any two indicators were presented in the heatmap, and the line graph focused on demonstrating the correlations between pgRNA and DNA, HBsAg, HBeAg. Asterisks indicate significance, **p < 0.01; ****p < 0.0001.

We compared the correlations between pgRNA and other indicators in pregnant (**Figure 4A**) and postpartum (**Figure 4B**) patients. The correlation coefficients between any two indicators were demonstrated using heatmaps, and the line graph focused on the correlations between pgRNA and DNA, HBsAg, and HBeAg. Positive correlations between RNA and DNA, HBsAg, and HBeAg were found in both the pregnancy and postpartum groups, with the highest correlation noted for pgRNA and HBeAg. We also described the relationship between pgRNA levels and markers of liver disease, and no correlation was found in either the pregnant or postpartum groups. In the pregnant group, a weak negative correlation between HBsAg and AFP (r = -0.36) was observed. In addition, a positive correlation between HBsAg and HBeAg (r = 0.59) and a positive correlation between DNA and HBsAg (r = 0.51) were noted in the pregnant group, but these correlations disappeared in the postpartum group.

### Factors influencing pgRNA levels in pregnant and postpartum patients with chronic hepatitis B

In the pregnant group (**Table 3**), univariate analysis revealed that antiviral treatment, HBsAg, HBeAg and AFP were the influencing factors for pgRNA levels. The OR of drug use was 6.199 (95% CI: 2.226 - 17.260) with a p-value of <0.001; the p-value of HBsAg was <0.001; the p-value of HBeAg was <0.001; and the p-value of AFP was 0.038.A further multifactorial analysis revealed that antiviral treatment was an independent influencing factor of pgRNA, with an OR of 5.321 (95% CI: 1.624 - 17.480) and a p-value of 0.006. Univariate analysis in the postpartum group revealed (**Table 4**) that antiviral treatment, HBsAg, and HBeAg were the influencing factors for pgRNA levels. The OR for drugs was 0.139 (95% CI: 0.048 - 0.402) with a p-value of <**0.001**, and the p-value for HBeAg was 0.031. Multifactorial analysis further revealed that drugs and HBeAg were independent predictors of pgRNA. The OR for drug use was 0.170 (95% CI: 0.035 - 0.821) with a p-value of 0.027; and the OR for HBeAg was 12.171 (95% CI: 1.337 - 110.783) with a p-value of 0.026. Notably, most pregnant patients receiving antiviral treatment have high DNA loads and positive HBeAg levels. For postpartum patients who have used antiviral treatment during pregnancy, continuous treatment is also needed after delivery. In essence, the DNA level and HBeAg affect pgRNA levels. Although antiviral treatment is correlated with high pgRNA levels, this relationship may be driven by the patient’s own relatively high DNA and HBeAg levels.

**Table 3:** Univariate and multivariate analyses of factors associated with pgRNA levels in the pregnant group.

Factors	Univariate analysis		Multivariate analysis	
	OR (95% CI)	p-value	OR (95% CI)	p-value
Age (years)	0.924 (0.839-1.016)	0.105	NA	
Weeks of pregnancy	0.990 (0.951-1.031)	0.648	NA	
Drug (yes vs. no)	6.199 (2.226-17.260)	**<0.001**	5.321 (1.624-17.480)	**0.006**
DNA (Log IU/ml)	1 (0.999-1)	0.060	NA	
HBsAg (IU/mL)	1 (1-1)	**<0.001**	1 (0.999-1.000)	0.105
HBeAg (COI)	1.002 (1-1.003)	**<0.001**	1 (0.999-1.000)	0.093
ALT (IU/L)	1.015 (0.985-1.046)	0.300	NA	
AST (IU/L)	1.007 (0.982-1.032)	0.557	NA	
AFP (ng/mL)	0.983 (0.968-0.999)	**0.038**	0.987 (0.969-1.004)	0.151
Four tests for liver fibrosis (normal vs. abnormal)	1.244 (0.454-3.409)	0.671	NA	

In the four indicators of liver fibrosis, if one or more items are positive, the patient was defined into the abnormal group.Bold values indicate p < 0.05.

**Table 4:** Univariate and multivariate analyses of factors associated with pgRNA levels in the postpartum group.

Factors	Univariate analysis		Multivariate analysis	
	OR (95% CI)	P value	OR (95% CI)	P value
Age (years)	1.008(0.944-1.078)	0.791	NA	
Month after postpartum (<12m vs.>12m)	0.685(0.292-1.606)	0.385	NA	
Drug (yes vs. no)	0.139(0.048-0.402)	**<0.001**	0.170(0.035-0.821)	**0.027**
DNA (Log IU/ml)	1(0.999-1)	0.249	NA	
HBsAg (IU/mL)	1(1-1)	**0.003**	1(0.999-1.000)	0.172
HBeAg (COI)	7.751(1.206-49.813)	**0.031**	12.171(1.337-110.783)	**0.026**
ALT(IU/L)	1.188(0.767-1.84)	0.439	NA	
AST(IU/L)	1.012(0.990-1.034)	0.274	NA	
AFP (ng/mL)	1.015(0.983-1.047)	0.351	NA	
Four tests for liver fibrosis (normal vs. abnormal)	2.938(0.772-11.181)	0.114	NA	

In the four indicators of liver fibrosis, if one or more items are positive, the patient was defined into the abnormal group.

## Discussion

Serum pgRNA, a surrogate marker of hepatic cccDNA transcriptional activity, has received increased attention in recent years (Kim et al., 2024). However, few studies have focused on the pregnant and postpartum patients with hepatitis B virus infection. In this study, we described the distribution of pgRNA levels in the pregnant and postpartum groups; verified the significant correlations between pgRNA and HBeAg, HBsAg, and HBV DNA; and further identified the factors affecting pgRNA positivity in the pregnant and postpartum groups. These findings highlighted the clinical significance of pgRNA.

In this study of pregnant and postpartum participants with CHB in eastern China, we first characterized the distribution of serum pgRNA. In both the pregnancy and postpartum groups, significant differences were observed in clinical indicators such as DNA, HBV antigen, ALT, AST, and AFP, however, there was no significant difference in pgRNA. These findings suggest that pgRNA levels may remain relatively stable across these physiological stages, reflecting persistent cccDNA activity despite changes in other virological and biochemical markers. Serum pgRNA levels were lower in HBeAg-negative patients than in HBeAg-positive patients. This is consistent with previous findings in smaller cohorts of patients (Maasoumy et al., 2015; Seto et al., 2014), and may be related to the reduced production of HBeAg after HBeAg seroconversion. Campenhout et al (van Campenhout et al., 2018)reported that HBeAg status is an independent factor associated with serum HBV pgRNA levels, as confirmed in the postpartum group of our research study. This might potentially account for the fact that the transcriptional activity of cccDNA is greater in HBeAg-positive patients than in HBeAg-negative patients. In addition, our study revealed that the use of antiviral therapy can affect pgRNA status. Patients receiving antiviral treatment are more likely to be positive for pgRNA during pregnancy and postpartum compared with those without regular antiviral treatment. On the one hand, the HBV pgRNA transcribed from HBV cccDNA may be degraded during the reverse transcription process. NUCs drugs can inhibit this reverse transcription process, leading to the accumulation of and increases HBV pgRNA in the serum of patients. On the other hand, according to the Prevention and Treatment Guidelines for Mother-to-Child Transmission of Hepatitis B Virus in China, pregnant women with high DNA loads or positive HBeAg levels must receive antiviral treatment. For postpartum patients who have used antiviral treatment during pregnancy, continuous treatment is also needed after delivery. In our cohort, a large proportion of patients experienced the above scenario. Thus, DNA and HBeAg levels affect pgRNA levels. Pan et al. (2021) reported that after 5 years of treatment with NUCs, serum HBV DNA was usually undetectable; however, 45% of patients were pgRNA positive, which could explain why most pregnant women infected with HBV experience virologic relapse after discontinuation of treatment postpartum. In the postpartum group, we further distinguished patients who received combined treatment with pegIFN-α and nucleoside/nucleotide analogues (NUCs) from and those who received NUCs alone. We found that pgRNA levels in patients who received combined treatment with pegIFN-α and NUCs after delivery were significantly lower compared with patients who received NUCs alone. This may be due to the killing effect of immune cells activated by pegIFN-α on infected liver cells, which clears some cells that are undergoing viral replication and contain a large amount of pgRNA, reducing the source of pgRNA.

Consistent with previous studies, we found stable associations between pgRNA and HBsAg, HBeAg, and HBV DNA levels in both the pregnant and postpartum groups (Liu et al., 2019; Mak et al., 2021; van Campenhout et al., 2018). Interestingly, a weak negative correlation between HBsAg and AFP was found in the pregnant group. AFP is elevated in normal pregnancies, mainly due to the synthesis of AFP by the fetal liver and yolk sac (Bartkute et al., 2017). However, a weak negative correlation occurs during pregnancy when the burden on the pregnant woman’s liver increases and hepatitis B virus infection may affect hepatocyte function, resulting in a relative decrease in AFP synthesis. Additionally, univariate analysis revealed that AFP was also an influential factor for pgRNA positivity in the pregnant group, suggesting that AFP has the potential to be used as an additional diagnostic tool, and that monitoring AFP levels, as well as pgRNA and other HBV markers, can provide a more comprehensive understanding of the disease status of pregnant women with chronic hepatitis B for timely intervention. In addition, a positive correlation was noted between HBsAg and HBeAg, HBsAg and DNA in the pregnant group; however, this correlation was weakened or even reversed in the postpartum group. This finding may be attributed to the fact that the replication of hepatitis B virus may be suppressed in the postpartum period with the restoration of hormone levels and adjustment of the immune system. In our study, we did not find a correlation between pgRNA and liver disease markers. In contrast, another North American study reported that HBV RNA levels were consistently positively correlated with markers of liver disease (ALT) in HBeAg-negative patients (Ghany et al., 2021). Li et al. reported a significant positive correlation of serum HBV RNA with ALT, AST, and DNA levels in patients with untreated HBeAg-positive and HBeAg-negative chronic hepatitis B infection (Li et al., 2018). The different populations of participants in the two studies, the differences in the prevalence of HBV genotypes, and the fact that pregnant women exhibit unique immune profiles could explain the different findings of the two studies. However, more studies are needed to confirm the correlation between pgRNA and liver disease markers.

To data, domestic and international basic and clinical studies have shown that HBV pgRNA has important clinical application value in the management of CHB, including predicting relapse after the discontinuation of antiviral therapy (Chen et al., 2018; Liu et al., 2020), guiding the duration of treatment (Ding et al., 2021; Wang F. D. et al., 2022), assessing disease progression (Liao et al., 2019), and serving as a target for new antiviral drugs (Vendeville et al., 2024). During pregnancy, pgRNA may further play a potential role. pgRNA levels reflect viral replication activity and helps predict the risk of mother-to-child transmission (MTCT). We retrieved the results of HBsAg and HBV DNA of the neonates at the time of birth and pgRNA levels of 73 pregnant CHB women (**Supplementary Table 1**). The data showed that only 4 neonates were positive for HBsAg at birth, but all of them turned negative for HBsAg two months after receiving the hepatitis B vaccine and hepatitis B immunoglobulin (HBIG). Due to the limitation of the follow-up time, we were unable to conduct further follow-up on the neonates who had completed the full course of the hepatitis B vaccine. However, based on the existing test results, the mother-to-child transmission blocking measures were generally successful. Future studies can further explore the relationship between the pgRNA level and the success rate of MTCT by expanding the sample size and extending the follow-up time. Second, pgRNA levels can guide the initiation and discontinuation of antiviral therapy during pregnancy. High pgRNA levels may indicate the need to initiate treatment earlier or delay discontinuation to reduce the risk of MTCT. In addition, pgRNA can also be used to evaluate the effectiveness of antiviral therapy. Dynamic monitoring of changes in pgRNA levels is helpful for optimizing the treatment plan.

To the best of our knowledge, this is the first study in China to visually compare the profiles and correlations of serum pgRNA levels in pregnant and postpartum populations with chronic HBV. However, our study has several limitations. Our study was based on real-life clinical retrospective practice, which inevitably suffers from retrospective bias. First, the sample size was relatively small, and more and larger prospective cohorts are needed to confirm the current findings. In addition, HBV genotypes were not detected. The HBV genotype is also an important factor affecting serum HBV RNA detection (Li et al., 2018). We described the phenotype without any hypothesis and further experiments are needed to determine the cause of this phenomenon. The predominant HBV genotypes in China are B and C (Yue et al., 2022), whereas A and D are the predominant genotypes in Europe (Demirel et al., 2024).Therefore, genotypes should also be considered in future studies to fully understand the relevance of the effect of the HBV genotype on serum pgRNA levels.

In summary, this study compared the distribution of pgRNA levels in chronic hepatitis B patients in the pregnant and postpartum groups, clarified the relationships between several laboratory indicators and pgRNA in different physiological stages, analyzed the factors affecting pgRNA positivity, and provided an important basis for an in-depth understanding of the pathophysiological characteristics of chronic hepatitis B during pregnancy and postpartum.

## Data Availability

The original contributions presented in the study are included in the article/**Supplementary Material**. Further inquiries can be directed to the corresponding author/s.

## Supplementary material

Supplementary Figure 1 pgRNA in pregnant and postpartum CHB patients with different levels of liver disease related markers. **(A)** A cohort of pregnant patients with hepatitis B virus were grouped by ALT (<40, n = 125,>40 = 9) and AST (<40, n = 131, >40, n = 3) level. **(B)** A cohort of postpartum patients with hepatitis B virus were grouped by ALT(<40, n = 88,>40 = 12), AST (<40, n = 93,>40 = 7), and AFP level (<10, n = 62; >10, n = 1). Then pgRNA levels were compared across the groups. The medians (interquartile range) are presented and statistical analyses were performed using Mann-Whitney test (M-W) between two group.

Supplementary Table 1 pgRNA levels in pregnant women with CHB and HBsAg, DNA levels in the neonates.
