# Supplementary figures and images for: A study on pregenomic RNA and factors in the pregnant and postpartum women with chronic HBV infection based on real world
Source: Front Cell Infect Microbiol. 2025 Apr 4;15:1539356. doi: 10.3389/fcimb.2025.1539356 (PMC12006107; doi:10.3389/fcimb.2025.1539356)

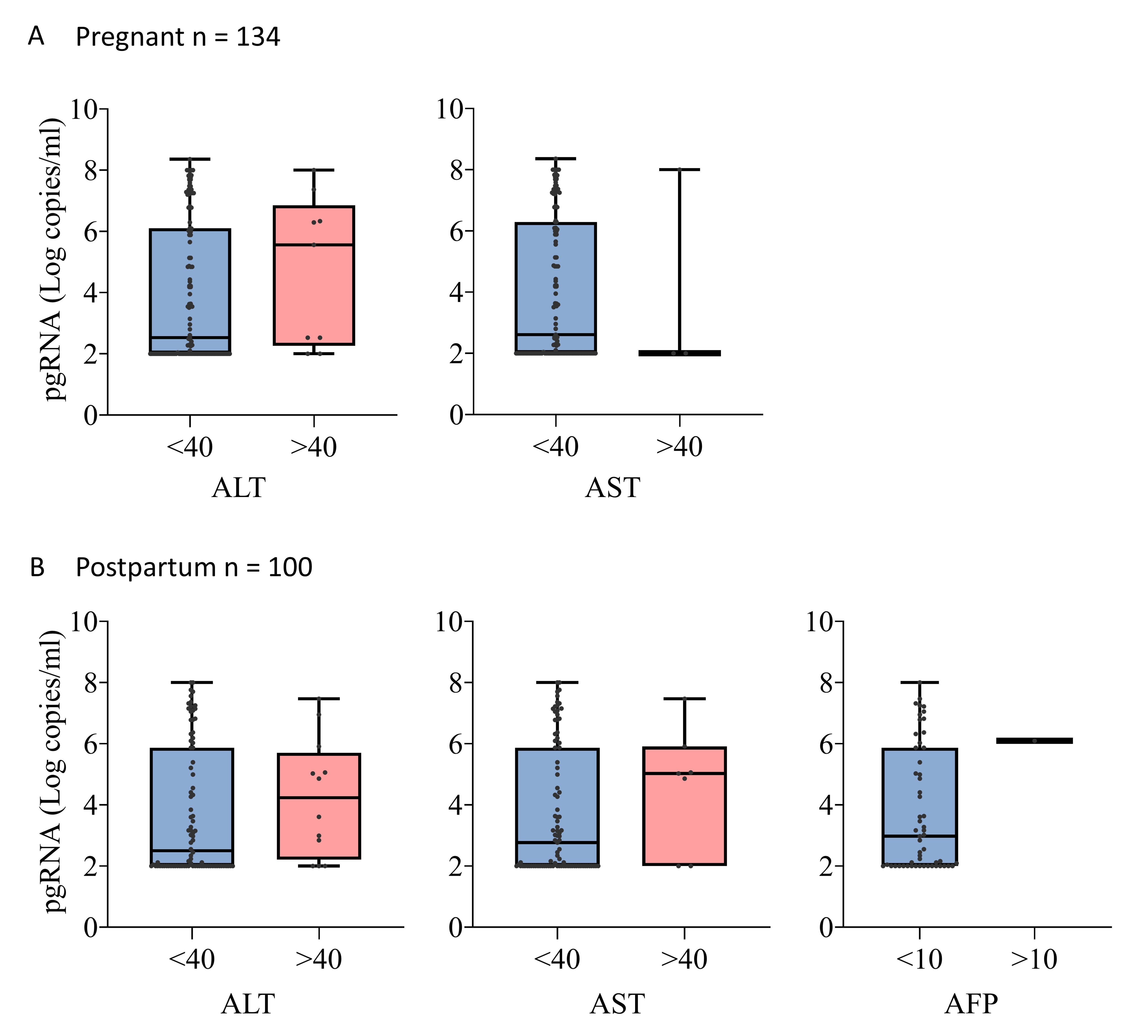

Supplement: Supplementary Figure 1 — pgRNA in pregnant and postpartum CHB patients with different levels of liver disease related markers. (A) A cohort of pregnant patients with hepatitis B virus were grouped by ALT (<40, n = 125,>40 = 9) and AST (<40, n = 131, >40, n = 3) level. (B) A cohort of postpartum patients with hepatitis B virus were grouped by ALT(<40, n = 88,>40 = 12), AST (<40, n = 93,>40 = 7), and AFP level (<10, n = 62; >10, n = 1). Then pgRNA levels were compared across the groups. The medians (interquartile range) are presented and statistical analyses were performed using Mann-Whitney test (M-W) between two group. [file Image1.jpeg]
