# Supplementary material for: A study on pregenomic RNA and factors in the pregnant and postpartum women with chronic HBV infection based on real world
Source: Front Cell Infect Microbiol. 2025 Apr 4;15:1539356. doi: 10.3389/fcimb.2025.1539356 (PMC12006107; doi:10.3389/fcimb.2025.1539356)
Supplement: Supplementary Table 1 — pgRNA levels in pregnant women with CHB and HBsAg, DNA levels in the neonates. [file Table1.docx]

**Supplementary Table 1. pgRNA levels in pregnant women with CHB and HBsAg, DNA levels in the neonates**

|  | Neonate | | Mother |
| --- | --- | --- | --- |
|  | HBsAg (IU/mL) | HBV DNA(IU/mL) | pgRNA(copies/mL) |
| Patient 1 | ＜0.05 | ＜500 | ＜100 |
| Patient 2 | 0 | ＜100 | ＜100 |
| Patient 3 | 0 | ＜100 | 112 |
| Patient 4 | 0 | ＜100 | 300 |
| Patient 5 | 0 | ＜100 | ＜100 |
| Patient 6 | 0 | ＜100 | ＜100 |
| Patient 7 | 0 | ＜20 | 337 |
| Patient 8 | 0.16 | ＜100 | 3960 |
| Patient 9 | 0 | ＜100 | ＜100 |
| Patient 10 | 0 | ＜100 | 337 |
| Patient 11 | 0 | ＜100 | ＜100 |
| Patient 12 | 0 | ＜100 | 15500 |
| Patient 13 | 0 | ＜100 | 3270 |
| Patient 14 | 0 | ＜100 | ＜100 |
| Patient 15 | 0 | ＜100 | ＜100 |
| Patient 16 | 0 | ＜100 | ＜100 |
| Patient 17 | 0 | ＜100 | ＜100 |
| Patient 18 | 0 | ＜100 | ＜100 |
| Patient 19 | 0 | ＜100 | ＜100 |
| Patient 20 | 0 | ＜100 | ＜100 |
| Patient 21 | 0 | ＜100 | ＜100 |
| Patient 22 | 0 | ＜100 | ＜100 |
| Patient 23 | 0 | ＜100 | 16500 |
| Patient 24 | 0 | ＜100 | ＜100 |
| Patient 25 | 0 | ＜100 | 1060000 |
| Patient 26 | 0 | ＜100 | 126 |
| Patient 27 | ＜0.05 | ＜500 | ＜100 |
| Patient 28 | 0 | ＜100 | 253 |
| Patient 29 | 0 | ＜100 | 194 |
| Patient 30 | 0 | ＜100 | ＜100 |
| Patient 31 | 0.12 | ＜100 | ＜100 |
| Patient 32 | 0.01 | ＜100 | ＜100 |
| Patient 33 | 0 | ＜100 | 202 |
| Patient 34 | 0 | ＜100 | ＜100 |
| Patient 35 | 0 | ＜100 | ＜100 |
| Patient 36 | 0 | ＜100 | 315 |
| Patient 37 | 0 | ＜100 | 23000 |
| Patient 38 | 0 | ＜100 | 4250 |
| Patient 39 | 0 | ＜100 | 73800 |
| Patient 40 | 0 | ＜100 | 767000 |
| Patient 41 | 0 | ＜100 | ＜100 |
| Patient 42 | 0 | ＜100 | 192 |
| Patient 43 | 0 | ＜100 | ＜100 |
| Patient 44 | 0 | ＜100 | 406 |
| Patient 45 | 0 | ＜100 | ＜100 |
| Patient 46 | 0 | ＜100 | 1970000 |
| Patient 47 | 0 | ＜100 | 1390 |
| Patient 48 | 0 | ＜100 | ＜100 |
| Patient 49 | ＜0.05 | ＜100 | 925 |
| Patient 50 | 0 | ＜100 | 19700000 |
| Patient 51 | ＜0.05 | ＜100 | 100000000 |
| Patient 52 | 0.01 | ＜100 | 18000000 |
| Patient 53 | 0 | ＜100 | 366000 |
| Patient 54 | ＜0.05 | ＜100 | 16600000 |
| Patient 55 | 0 | ＜20 | 1970000 |
| Patient 56 | 0 | ＜100 | ＜100 |
| Patient 57 | 0.03 | ＜500 | 49900000 |
| Patient 58 | ＜0.05 | ＜500 | ＜100 |
| Patient 59 | 0 | ＜20 | 6020000 |
| Patient 60 | 0 | ＜100 | 75000000 |
| Patient 61 | 0.01 | ＜500 | 70300 |
| Patient 62 | 0.18 | ＜100 | 100000000 |
| Patient 63 | 0.08 | ＜100 | 690000000 |
| Patient 64 | 0 | ＜100 | 2180000 |
| Patient 65 | 0 | ＜500 | ＜100 |
| Patient 66 | 0 | ＜100 | 997000 |
| Patient 67 | 0 | ＜100 | 6600000 |
| Patient 68 | 0.03 | ＜20 | 30000000 |
| Patient 69 | 0 | ＜100 | 15900000 |
| Patient 70 | 0 | ＜100 | 30500000 |
| Patient 71 | 0 | ＜100 | 58000000 |
| Patient 72 | 0.31 | ＜500 | 1280000 |
| Patient 73 | 0 | ＜100 | 1130000 |
